# Serum 25-hydroxyvitamin D mediates the association between heavy metal exposure and cardiovascular disease

**DOI:** 10.1186/s12889-024-18058-z

**Published:** 2024-02-21

**Authors:** Yan Lu, Licheng Lu, Gang Zhang, Weiguo Zhang, Yazhuo Cheng, Mingyue Tong

**Affiliations:** 1 Department of Cardiology, The People’s Hospital of Suzhou New District, No.95 Huashan Road, Suzhou High-Tech Zone, Suzhou, Jiangsu 215129 China; 2 Department of Cardiology, Kunshan Hospital of Traditional Chinese Medicine, No.388 Zuchongzhi Road, Kunshan, Jiangsu 215300 China; 3 Department of Rehabilitation, The First Affiliated Hospital of Anhui Medical University, 100 Huaihai Dadao, Xinzhan District, Hefei, Anhui 230000 China; 4 Department of Rehabilitation, Anhui Public Health Clinical Center, 100 Huaihai Dadao, Xinzhan District, Hefei, Anhui 230000 China

**Keywords:** Cardiovascular disease, Heavy metals exposure, Mediation analysis, Serum 25-hydroxyvitamin D, United States

## Abstract

**Background:**

Mediation analysis aims to determine how intermediate variables affect exposure to disease. In this study, 25-hydroxyvitamin D (25(OH)D) was evaluated to assess its role in mediating heavy metal exposure and cardiovascular disease (CVD).

**Methods:**

A total of 9,377 participants from the National Health and Nutrition Examination Survey (NHANES) for the years 2011-2018 were included. Firstly, restricted cubic spline (RCS), and multivariable logistic regression model were performed to estimate the association between heavy metal exposure (Cadmium, Lead, Mercury, Manganese, and Selenium), as well as serum 25(OH)D and CVD. Secondly, using generalized linear regression model and generalized additive models with smooth functions, we investigated the correlation between heavy metal exposure and serum 25(OH)D. Finally, the mediation effect of serum 25(OH)D in the associations between heavy metal exposure and CVD was explored.

**Results:**

The RCS plots revealed that Cadmium, and Lead were positively and linearly associated with CVD, while Mercury, and Manganese were inversely and linearly associated with CVD. Additionally, a roughly L- and U-shaped relationship existed between Selenium, as well as 25(OH)D and CVD. When potential confounding factors were adjusted for, serum 25(OH)D had negative associations with Cadmium, Lead, and Manganese, while serum 25(OH)D had positive relationship with Selenium. There was a mediation effect between Manganese exposure and CVD, which was mediated by 25(OH)D.

**Conclusion:**

According to the mediation analysis, the negative association between Manganese exposure and incident CVD was increased by 25(OH)D. The increasing dietary intake of Vitamin D could increase the protective effect of manganese intake on CVD.

## Introduction

In the world, cardiovascular disease (CVD) account for nearly one-third of all deaths, making them an important public health problem [1]. The top six causes of CVD death include hypertension, smoking, diabetes mellitus or elevated glucose level, elevated cholesterol levels, and obesity or being overweight [2]. However, there is increasing evidence that the incidence of CVD appears to also be linked to environmental pollutants including polyfluoroalkyl chemicals, acrylamide, and so on [3–5]. As a result of their extensive use in industries, homes, agriculture, and medicine, heavy metals are widely distributed throughout the environment [6]. It is reported that most heavy metals are highly toxic [7]. Besides ingestion and inhalation, they can also be absorbed through the skin, causing heavy health effects when in contact with humans [8, 9]. Arsenic and other (often co-occurring) toxic metals have also been suggested as independent cardiovascular disease risk factors [10, 11]. Vitamin D plays a crucial role in calcium and phosphorus metabolism in bones and minerals as a fat-soluble vitamin [12]. Besides its well-known role in calcium homeostasis, Vitamin D deficiency also contributes to CVD [13]. A new study revealed that the complicated relationship between vitamin D, folate, and heavy metals may be able to lessen the impact on blood pressure [14]. It is not known in the United States (U.S.) general population whether serum 25-hydroxyvitamin D (25(OH)D) levels are related to heavy metal exposure. The research also shows that the metabolism of heavy metals is affected by the Vitamin D status of the chicken body [15]. According to our hypothesis, 25(OH)D regulates heavy metal metabolism, thus reducing the harmful effects of heavy metal exposure. Therefore, in this study, we investigated the link between heavy metal exposure and 25(OH)D using the National Health and Nutrition Examination Survey (NHANES) database. Additionally, the mediation effect of vitamin D on heavy metal exposure in humans with CVD was further explored for the first time.

## Material and methods

### Study population

**Fig. 1:**
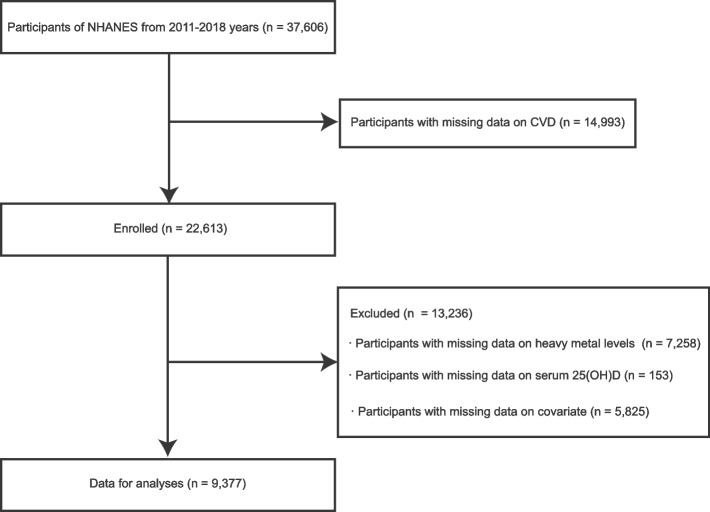
Study flow chart. Abbreviations: NHANES, National Health and Nutrition Examination Surveys; CVD, cardiovascular disease; serum 25(OH)D, serum 25-hydroxyvitamin D

The NHANES cross-sectional database contains multitudinous data about the nutrition and health of the general U.S. population based on a dynamic, multistage probability sampling design [16]. The NHANES data from four cycle years (2011 to 2018) were used to examine whether serum 25(OH)D mediates the relationship between heavy metal exposure and cardiovascular disease. A total of 14,993 participants without CVD data were removed from the 37,606 total participants in the sample. A further exclusion was made for participants who were missing data on heavy metals (*n* = 7,258) and serum 25(OH)D (*n* = 153). In addition, we also removed the patient’s missing demographic and biochemical data to ensure the accuracy of the results (*n* = 5,825). Finally, our analysis included 9,377 participants in total (Fig. 1). The NHANES website provides detailed information about the survey design, methodology, and data (https://www.cdc.gov/nchs/nhanes/).

### Heavy metals measurements

After participants were verified to be free of background contamination, a blood sample was taken from them. The National Center for Environmental Health, and the Centers for Disease Control and Prevention, Atlanta, GA, receive and process whole blood samples for analysis. Whole blood samples were analyzed for Cadmium, Lead, Mercury, Manganese, and Selenium content using mass spectrometry. The details of the experiment can be found here https://wwwn.cdc.gov/nchs/data/nhanes/labmethods/PBCD_I_met.pdf.

### CVD ascertainment

In the study, the outcomes of CVD included coronary heart disease (CHD), congestive heart failure (CHF), angina pectoris, heart attack, and stroke [17]. Participants were asked to complete a questionnaire to determine whether they had new or pre-existing diseases during this period. Qualified interviewers administered a standardized medical condition questionnaire to participants: “Have you ever been told that you have congestive heart failure/coronary heart disease/angina pectoris/heart attack/stroke?” There is more information about the questionnaire at https://wwwn.cdc.gov/nchs/nhanes/search/datapage.aspx?Component=Questionnaire.

### Covariates

A range of covariates associated with CVD outcome was identified and controlled after heavy metal exposure was evaluated. The following covariates were taken into account in the analysis: demographic data (age, sex (man/women), race/ethnicity (Mexican American, other Hispanic American, non-Hispanic black, non-Hispanic white, and other races), education level (less than high school/high school/more than high school), family poverty-income ratio (PIR), marital status (have a partner/no partner/unmarried)), smoking and alcohol consumption (smoker (never/former/now), alcohol user (never/former/mild/moderate/heavy)), medical history (the history of hypertension, and diabetes mellitus (DM)), and body mass index (BMI), waist circumference, and mean energy intake. In addition, laboratory tests provided information on hemoglobin (Hb), high-density lipoprotein cholesterol (HDL-C), total cholesterol (TC), triglyceride (TG), blood urea nitrogen (BUN), uric acid (UA), serum creatinine (Scr), and serum 25(OH)D. You can find more information about the NHANES procedures here https://wwwn.cdc.gov/nchs/nhanes/Default.aspx.

### Statistical analysis

Our study utilized R version 3.6.4 (R Foundation for Statistical Computing, Vienna, Austria), and SPSS version 22.0 (SPSS Inc., Chicago, IL, USA) for statistical analysis. Based on the NHANES sample weights, all estimates were calculated. The continuous variables described by using the means ± standard deviation (SD) and the categorical variables were expressed as number (percentage, %). The differences between different groups were calculated using weighted linear regression models (continuous variables) or weighted chi-square tests (categorical variables). Due to its skewed distribution, heavy metals and 25(OH)D concentration was transformed using log10 transformation. The association between heavy metals exposure, as well as 25(OH)D, and the incidence of CVD was studied using restricted cubic spline (RCS) and multivariable logistic regression models. In addition, to evaluate the relationship between the 25(OH)D level and the levels of heavy metals, generalized linear regression models and generalized additive models with smooth functions were performed. The RCS, multivariable logistic regression, and generalized linear regression model adjusted the following covariates: Model 1 was adjusted for age and sex. In model 2, we further adjusted for other potential confounders, including race/ethnicity, education level, material status, family PIR, smoker, alcohol user, the history of hypertension, and DM. On the basis of Model 2, comprehensive adjustments for BMI, waist circumference, mean energy intake, TC, TG, HDL-C, BUN, UA, and Scr were made (Model 3). In mediation analysis, a mediating variable (M) is assumed to mediate the relationship between independent variables (X) and dependent variables (Y) [18]. An indirect effect (IE) and a direct effect (DE) of heavy metal exposure on CVD were evaluated separately. The independent variable was heavy metals exposure (X), the outcome variable was CVD (Y), and the mediating variable was the level of 25(OH)D (M). An analysis of mediation was carried out to determine if the serum vitamin D level mediated the association between heavy metal exposure and CVD. CVD was affected by heavy metals exposure as measured by TE. The IE refers to the effect of heavy metal exposure on CVD through the level of 25(OH)D. DE represents the effect of heavy metal exposure on CVD when 25(OH)D is controlled for. To reflect the proportion of mediation by 25(OH)D, the proportion of IE in TE was calculated. Mediation analysis adjusted for age, sex, race/ethnicity, educational level, marital status, family PIR, smoker, alcohol user, the history of hypertension, and DM, BMI, waist circumference, mean energy intake, Hb, TC, TG, HDL-C, BUN, UA, and Scr as a covariate. There may be serious collinearity problems when five heavy metals are entered into the model at the same time. Therefore, we further used gWQS regression to fit models to explore the total effect of mixed exposure to the five heavy metals and the relative contribution of each factor [19]. *P*-value < 0.05 was considered statistically significant.

## Results

### Baseline characteristics

Our study involved a total of 9,377 participants. The weighted demographic and medical characteristics were listed in Table 1. The incidence of CVD was found to be present in 9.6% of the study population. Participants were significantly different in terms of age, sex, different ethnic composition, UA, family PIR, smoker, alcohol user, the complication of hypertension, BMI, TG, marital status, Manganese, mean energy intake, Hb, education level, BUN, Mercury, Scr, TC, HDL-C, and Selenium when grouping by vitamin D quartiles.

**Table 1:** Weighted characteristics of the study population based on serum 25(OH)D quartiles

Vitamin D (nmol/L)	Total	Q1	Q2	Q3	Q4	*P*-value
Age, years	47.75 ± 0.44	42.36 ± 0.49	43.15 ± 0.60	47.25 ± 0.54	54.60 ± 0.52	< 0.001
Sex, %						
Male	4630 (49.4%)	1152 (12.3%)	1290 (13.8%)	1211 (12.9%)	977 (10.4%)	< 0.001
Female	4747 (50.6%)	1199 (12.8%)	1056 (11.3%)	1130 (12.1%)	1362 (14.5%)	
Race, %						< 0.001
Mexican American	1115 (11.9%)	369 (3.9%)	358 (3.8%)	259 (2.8%)	129 (1.4%)	
Other Hispanic	927 (9.9%)	194 (2.1%)	317 (3.4%)	253 (2.7%)	163 (1.7%)	
Non-Hispanic Black	2096 (22.4%)	972 (10.4%)	480 (5.1%)	330 (3.5%)	314 (3.3%)	
Non-Hispanic White	3837 (40.9%)	419 (4.5%)	819 (8.7%)	1154 (12.3%)	1445 (15.4%)	
Other race	1402 (15.0%)	397 (4.2%)	372 (4.0%)	345 (3.7%)	288 (3.1%)	
Family PIR	3.11 ± 0.05	2.53 ± 0.07	2.89 ± 0.06	3.24 ± 0.07	3.46 ± 0.06	< 0.001
Education level, %						< 0.001
Less than high school	1663 (17.7%)	435 (4.6%)	474 (5.1%)	397 (4.2%)	357 (3.8%)	
High school	2073 (22.1%)	569 (6.1%)	537 (5.7%)	498 (5.3%)	469 (5.0%)	
More than high school	5641 (60.2%)	1347 (14.4%)	1335 (14.2%)	1446 (15.4%)	1513 (16.1%)	
Marital status, %						< 0.001
Having a partner	5581 (59.5%)	1223 (13.0%)	1387 (14.8%)	1501 (16.0%)	1470 (15.7%)	
No partner	1953 (20.8%)	436 (4.6%)	440 (4.7%)	471 (5.0%)	606 (6.5%)	
Unmarried	1843 (19.7%)	692 (7.4%)	519 (5.5%)	369(3.9%)	263 (2.8%)	
Smoker, %						< 0.001
Never	5395 (57.5%)	1389 (14.8%)	1398 (14.9%)	1325 (14.1%)	1283 (13.7%)	
Former	2232 (23.8%)	419 (4.5%)	493 (5.3%)	600 (6.4%)	720 (7.7%)	
Now	1750 (18.7%)	543 (5.8%)	455 (4.9%)	416 (4.4%)	336 (3.6%)	
Alcohol user, %						< 0.001
No	1224 (13.1%)	350 (3.7%)	314 (3.3%)	254 (2.7%)	306 (3.3%)	
Former	1113 (11.9%)	231 (2.5%)	289 (3.1%)	277 (3.0%)	316 (3.4%)	
Mild	3565 (38.0%)	788 (8.4%)	842 (9.0%)	948 (10.1%)	986 (10.5%)	
Moderate	1594 (17.0%)	431 (4.6%)	375 (4.0%)	421 (4.5%)	367 (3.9%)	
Heavy	1882 (20.1%)	551 (5.9%)	526 (5.6%)	441 (4.7%)	364 (3.9%)	
Hypertension, %						< 0.001
No	5358 (57.1%)	1415 (15.1%)	1478 (15.8%)	1377 (14.7%)	1088 (11.6%)	
Yes	4019 (42.9%)	936 (10.0%)	868 (9.3%)	964 (10.3%)	1251 (13.3%)	
DM, %						0.063
No	7632 (81.4%)	1903 (20.3%)	1942 (20.7%)	1937 (20.7%)	1850 (19.7%)	
Yes	1745 (18.6%)	448 (4.8%)	404 (4.3%)	404 (4.3%)	489(5.2%)	
CHD, %						0.046
No	9032 (96.3%)	2301 (24.5%)	2272 (24.2%)	2250 (24.0%)	2209 (23.6%)	
Yes	345 (3.7%)	50 (0.5%)	74 (0.8%)	91 (1.0%)	130 (1.4%)	
CHF, %						0.001
No	9109 (97.1%)	2287 (24.2%)	2286 (24.4%)	2295 (24.5%)	2241 (23.9%)	
Yes	268 (2.9%)	64 (0.7%)	60 (0.6%)	46 (0.5%)	98 (1.0%)	
Angina pectoris, %						0.479
No	9165 (97.7%)	2306 (24.6%)	2297 (24.5%)	2297 (24.5%)	2265 (24.2%)	
Yes	212 (2.3%)	45 (0.5%)	49 (0.5%)	44 (0.5%)	74 (0.8%)	
Heart attack, %						0.143
No	9020 (96.2%)	2278 (24.3%)	2270 (24.2%)	2264 (24.1%)	2208 (23.5%)	
Yes	357 (3.8%)	73 (0.8%)	76 (0.8%)	77 (0.8%)	131 (1.4%)	
Stroke, %						< 0.001
No	9038 (96.4%)	2276 (24.3%)	2288 (24.4%)	2261 (24.1%)	2213 (23.6%)	
Yes	339 (3.6%)	75 (3.2%)	58 (0.6%)	80 (0.9%)	126 (1.3%)	
CVD, %						< 0.001
No	8473 (90.4%)	2165 (23.1%)	2163 (23.1%)	2134 (22.8%)	2011 (21.4%)	
Yes	904 (9.6%)	186 (2.0%)	183 (2.0%)	207 (2.2%)	328 (3.5%)	
BMI, kg/m^2^	29.35 ± 0.14	31.69 ± 0.21	30.00 ± 0.25	29.00 ± 0.19	27.88 ± 0.19	< 0.001
Mean energy	2109.93 ± 13.11	2137.60 ± 23.82	2138.54 ± 24.69	2150.32 ± 21.81	2037.33 ± 19.35	< 0.001
Intake (kcal/day)						
Hemoglobin, g/dL	14.23 ± 0.03	14.05 ± 0.06	14.37 ± 0.05	14.33 ± 0.04	14.14 ± 0.04	< 0.001
BUN, mg/dL	13.91 ± 0.09	12.09 ± 0.15	13.42 ± 0.15	14.03 ± 0.15	15.20 ± 0.16	< 0.001
UA, mg/dL	5.42 ± 0.02	5.52 ± 0.04	5.52 ± 0.04	5.41 ± 0.04	5.30 ± 0.04	< 0.001
Scr, mg/dL	0.88 ± 0.00	0.85 ± 0.01	0.87 ± 0.01	0.88 ± 0.00	0.92 ± 0.01	< 0.001
TC, mg/dL	193.25 ± 0.78	190.15 ± 1.22	189.77 ± 1.20	194.48 ± 1.06	196.44 ± 1.35	< 0.001
TG, mg/dL	150.15 ± 2.06	155.64 ± 4.43	157.13 ± 3.90	151.17 ± 3.56	141.02 ± 2.81	< 0.001
HDL-cholesterol, mg/dL	53.76 ± 0.33	50.49 ± 0.38	49.91 ± 0.47	53.20 ± 0.43	58.92 ± 0.59	< 0.001
Cadmium, ug/L	0.44 ± 0.01	0.49 ± 0.02	0.45 ± 0.02	0.42 ± 0.02	0.44 ± 0.02	0.093
Lead, ug/dL	1.24 ± 0.03	1.23 ± 0.06	1.19 ± 0.05	1.23 ± 0.04	1.28 ± 0.04	0.381
Mercury, ug/L	1.40 ± 0.07	1.03 ± 0.05	1.33 ± 0.10	1.53 ± 0.07	1.53 ± 0.10	< 0.001
Manganese, ug/L	9.71 ± 0.05	10.12 ± 0.11	9.93 ± 0.13	9.55 ± 0.09	9.46 ± 0.07	< 0.001
Selenium, ug/L	195.90 ± 0.80	193.69 ± 0.99	194.93 ± 0.93	196.12 ± 0.92	197.65 ± 1.26	0.022

Data are presented as mean ± (standard deviation, SD) or n (%)

Q1, 7.83–45.8 ng/ml; Q2, 45.9–63.2 ng/ml; Q3 63.3–82.4 ng/ml; Q4 82.3–422 ng/L

*Abbreviations*: *Serum 25(OH)D* 25-hydroxyvitamin D, *DM* diabetes mellitus, *CHD* coronary heart disease, *CHF* congestive heart failure, *CVD* cardiovascular disease, *BMI* body mass index, *TC* total cholesterol, *TG* triglyceride, *HDL-C* high density lipoprotein- cholesterol

### Mixture effect between heavy metals

**Fig. 2:**
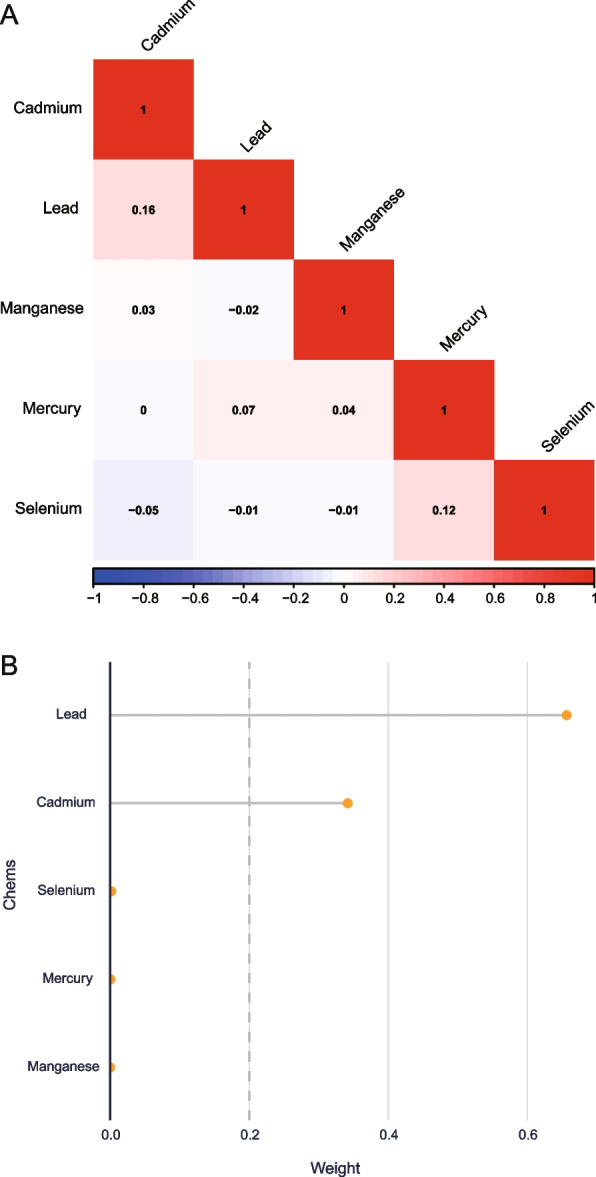
**A** Correlation heat map of the five heavy metal relationships; **B** Weight of exposure effect of five heavy metals on cardiovascular events

We found that Cadmium was positively related with Lead (*r* = 0.16), Mercury was positively related with Selenium (*r* = 0.12), and Lead was positively related with Mercury (*r* = 0.07) by correlation coefficient heat map (Fig. 2A). The regression coefficient of WOS index was 0.575 (*P* < 0.001), reflecting the total mixed exposure effect of exposure to five heavy metals. The overall effect of the five heavy metals exposure were positively and significantly associated with CVD outcomes. Among them, Lead and Cadmium contributed the most to the total effect of exposure (Fig. 2B).

### Associations between heavy metals, as well as 25(OH)D and CVD

**Fig. 3:**
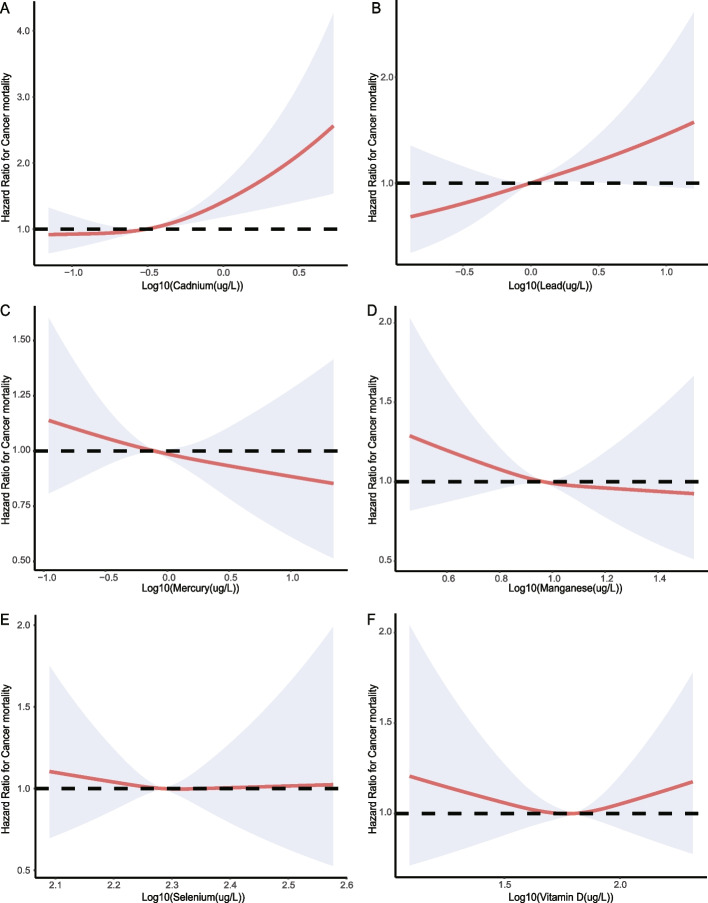
The restricted cubic spline plots of associations of heavy metals (Cadmium, Lead, Mercury, Manganese, and Selenium) and serum 25(OH)D with prevalence of CVD. Note: Heavy metals, and 25(OH)D are log10 transformed. Analyses were adjusted for age, sex, race /ethnicity, education level, family poverty-income ratio, the history of hypertension, diabetes mellitus, smoking, alcohol use, body mass index, mean energy intake, hemoglobin, high-density cholesterol, triglycerides, cholesterol, blood urea nitrogen, serum creatinine, uric acid. The solid and dashed lines represent the log-transformed odds ratios and the corresponding 95% confidence intervals. Abbreviations: 25(OH)D, 25-hydroxyvitamin D

The RCS plot curve is shown in Fig. 3A, and Fig. 3B, representing the positive and linear correlation between Cadmium, as well as Lead and the incidence of CVD (*P* for nonlinearity > 0.05). In addition, an inverse and linear association existed between Mercury, as well as Manganese and CVD. (Fig. 3C, and D, *P* for nonlinearity > 0.05). The connection between Selenium, as well as 25(OH)D, and CVD was approximately L- and U-shaped (Fig. 3E, and F), respectively. Additionally, the findings of multivariate logistic regression analysis of the association of heavy metals, and serum 25(OH)D with prevalence of CVD was presented in Table 2.

**Table 2:** Adjusted ORs for associations of heavy metals, and serum 25(OH)D with prevalence of CVD

Variable	Model 1		Model 2		Model 3	
	OR (95%CI)	*P* for trend	OR (95%CI)	*P* for trend	OR (95%CI)	*P* for trend
Cadmium (ug/L)		< 0.001		0.035		0.004
Q1	1.00		1.00		1.00	
Q2	0.929 (0.731, 1.182)		0.951 (0.743, 1.217)		0.998 (0.776, 1.284)	
Q3	1.121 (0.888, 1.414)		1.067 (0.836, 1.362)		1.152 (0.896, 1.480)	
Q4	1.815 (1.454, 2.266)^***^		1.332 (1.022, 1.736)^*^		1.513 (1.148, 1.994)^**^	
Lead (ug/L)		0.536		0.697		0.138
Q1	1.00		1.00		1.00	
Q2	1.120 (0.847, 1.482)		1.105 (0.829, 1.473)		1.238 (0.925, 1.658)	
Q3	1.056 (0.802, 1.390)		1.015 (0.764, 1.348)		1.213 (0.907, 1.624)	
Q4	1.180 (0.897, 1.552)		1.117 (0.837, 1.489)		1.409 (1.043, 1.905)^*^	
Mercury (ug/L)		< 0.001		0.149		0.611
Q1	1.00		1.00		1.00	
Q2	0.864 (0.706, 1.059)		0.938 (0.761, 1.155)		0.949 (0.767, 1.173)	
Q3	0.739 (0.600, 0.910)^**^		0.883 (0.712, 1.095)		0.936 (0.750, 1.166)	
Q4	0.603 (0.488, 0.745)^***^		0.777 (0.622, 0.970)^*^		0.855 (0.680, 1.076)	
Manganese (ug/L)		0.126		0.336		0.446
Q1	1.00		1.00		1.00	
Q2	0.859 (0.707, 1.045)		0.933 (0.763, 1.142)		0.980 (0.796, 1.206)	
Q3	0.885 (0.725, 1.080)		0.931 (0.758, 1.143)		0.984 (0.797, 1.215)	
Q4	0.777 (0.627, 0.962)^*^		0.813 (0.652, 1.014)		0.841 (0.670, 1.055)	
Selenium (ug/L)		0.016		0.132		0.507
Q1	1.00		1.00		1.00	
Q2	0.768 (0.627, 0.940)^*^		0.807 (0.655, 0.995)^*^		0.861 (0.695, 1.068)	
Q3	0.799 (0.652, 0.978)^*^		0.891 (0.723, 1.099)		0.994 (0.800, 1.235)	
Q4	0.747 (0.609, 0.916)^**^		0.805 (0.652, 0.994)^*^		0.969 (0.775, 1.211)	
Serum 25(OH)D		0.002		0.308		0.570
Q1	1.00		1.00		1.00	
Q2	0.752 (0.599, 0.945)^*^		0.886 (0.701, 1.120)		0.934 (0.735, 1.188)	
Q3	0.644 (0.515, 0.805)^***^		0.813 (0.645, 1.025)		0.871 (0.687, 1.105)	
Q4	0.744 (0.602, 0.919)^**^		0.942 (0.754, 1.176)		0.996 (0.791, 1.255)	

Model 1: age and sex

Model 2: model 1 variables plus race/ethnicity, education level, marital status, family poverty-income ratio, smoker, alcohol user, hypertension, and diabetes mellitus. Model 3 was adjusted for model 2 variables plus body mass index, mean energy intake, hemoglobin, blood urea nitrogen, uric acid, creatinine, high density lipoprotein-cholesterol, total cholesterol, and triglycerides

Cadmium quartile (Q1: 0.07–0.19 ug/L; Q2: 0.20–0.31 ug/L; Q3: 0.32–0.55 ug/L; and Q4: 0.56–13.03 ug/L); Lead quartile (Q1: 0.05–0.63 ug/L; Q2: 0.64–1.00 ug/L; Q3: 1.01–1.59 ug/L; and Q4: 1.60–61.29 ug/L); Mercury quartile (Q1: 0.11–0.41 ug/L; Q2: 0.42–0.79 ug/L; Q3: 0.80–1.62 ug/L; and Q4: 1.63–50.81 ug/L); Manganese quartile (Q1:1.57–7.43 ug/L; Q2: 7.44–9.24 ug/L; Q3: 9.25–11.55 ug/L; and Q4: 11.56–57.77 ug/L); Selenium quartile (Q1: 85.15–177.87 ug/L; Q2: 177.88–192.47 ug/L; Q3: 192.48–207.54 ug/L; and Q4: 207.55–734.80 ug/L)

*Abbreviations*: *Serum 25(OH)D* 25-hydroxyvitamin D, *CVD* cardiovascular disease, *OR* odd ratio, *CI* confidence interval

^*^P < 0.05

^**^P < 0.01

^***^P < 0.001

### Association of serum 25(OH)D with heavy metals levels

**Fig. 4:**
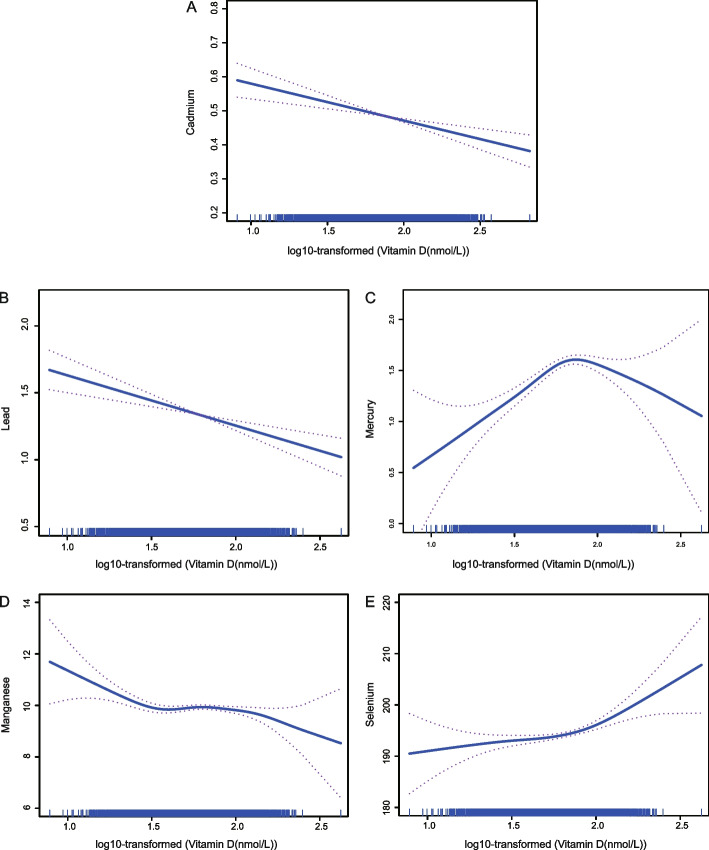
The association of serum 25(OH)D with heavy metals. **A** The association between 25(OH)D and Cadmium; **B** The association between 25(OH)D and Lead; **C** The association between 25(OH)D and Mercury; **D** The association between 25(OH)D and Manganese; **E** The association between 25(OH)D and Selenium. Abbreviations: 25(OH)D, 25-hydroxyvitamin D

The multivariate generalized linear regression coefficients of the content of heavy metals with increasing 25(OH)D levels are shown in Table 3. There was a consistent association between heavy metal levels and 25(OH)D levels in all three models (Model 1, Model 2, and Model 3). In the full adjusted model, compared with the Q1, the 25(OH)D levels in the Q4 was independently associated with the level of Cadmium (β =  − 15.81 × 10^−2^, *P* for trend < 0.001), Lead (β =  − 9.20 × 10^−2^, *P* for trend < 0.001), Mercury (β = 0.11 × 1^−2^, *P* for trend = 0.526), Manganese (β =  − 1.50 × 10^−2^, *P* for trend = 0.287), and Selenium (β = 2.25 × 10^−2^, *P* for trend = 0.133). Generalized additive models with smooth functions showed the 25(OH)D levels to have a negative and linear association with the level of Cadmium, Lead, Mercury, as well as Manganese, and a positive and linear association with the Selenium level (Fig. 4A, B, D, and E). However, there was an increase and then a decrease in the relationship between 25(OH)D levels and Mercury (Fig. 4C).

**Table 3:** Association between serum 25(OH)D and heavy metals

Vitamin D, nmol/L	Model 1	Model 2	Model 3
	β (95% CI) *10^−2^	β (95% CI) *10^−2^	β (95% CI) *10^−2^
Cadmium			
Q1	Ref	Ref	Ref
Q2	-3.27 (-3.98, -2.57)^***^	-4.30 (-5.02, -3.59)^***^	-4.79 (-5.50, -4.08)^***^
Q3	-5.65 (-6.38, -4.92)^***^	-8.00 (-8.75, -7.24)^***^	-9.83 (-10.63, -9.11)^***^
Q4	-7.90 (-8.62, -7.18)^***^	-11.70 (-12.56, -10.83)^***^	-15.81 (-16.69, -14.92)^***^
*P* for trend	< 0.001	< 0.001	< 0.001
Lead			
Q1	Ref	Ref	Ref
Q2	1.50 (0.76, 2.24)^***^	0.43 (-0.31, 1.18)	-1.44 (-2.91, -0.69)^***^
Q3	-2.25 (-1.00, 0.55)	-1.87 (-2.66, -1.09)^***^	-4.41 (-5.22, -3.62)^***^
Q4	-2.65 (-3.47, -1.84)^***^	-4.47 (-5.31, -3.63)^***^	-9.20 (-10.07, -8.33)^***^
*P* for trend	0.016	< 0.001	< 0.001
Mercury			
Q1	Ref	Ref	Ref
Q2	2.72 (2.01, 3.44)^***^	2.86 (2.14, 3.58)^***^	2.60 (1.89, 3.32)^***^
Q3	2.70 (1.98, 3.42)^***^	1.22 (0.50, 1.95)^***^	0.05 (-0.68, 0.78)
Q4	8.42 (7.71, 9.12)^***^	3.16 (2.43, 3.89)^***^	0.11 (-0.63, 0.86)
*P* for trend	0.005	0.002	0.001
Manganese			
Q1	Ref	Ref	Ref
Q2	3.68 (2.97, 4.37)^***^	2.83 (2.13, 3.53)^***^	2.76 (2.06, 3.47)^***^
Q3	3.04 (2.34, 3.75)^***^	2.20 (1.49, 2.91)^***^	2.21 (1.49, 2.92)^***^
Q4	-0.98 (-1.71, -0.26)^**^	-2.21 (-2.94, -1.48)^***^	-1.50 (-2.23, -7.62)^***^
*P* for trend	0.001	0.001	< 0.001
Selenium			
Q1	Ref	Ref	Ref
Q2	2.63 (1.92, 3.33)^***^	1.57 (0.86, 2.27)^***^	1.01 (0.29, 1.72)^**^
Q3	2.30 (1.59, 3.01)^***^	0.81 (0.09, 1.51)^*^	-0.19 (-0.91, -0.53)
Q4	5.54 (4.84, 6.25)^***^	3.71 (3.00, 4.42)^***^	2.25 (1.52, 2.99)^***^
*P* for trend	0.006	0.003	< 0.001

Model 1: age and sex

Model 2: model 1 variables plus race/ethnicity, education level, marital status, family poverty-income ratio, smoker, alcohol user, hypertension, and diabetes mellitus. Model 3 was adjusted for model 2 variables plus body mass index, mean energy intake, hemoglobin, blood urea nitrogen, uric acid, creatinine, high density lipoprotein-cholesterol, total cholesterol, and triglycerides

Cadmium quartile (Q1: 0.07–0.19 ug/L; Q2: 0.20–0.31 ug/L; Q3: 0.32–0.55 ug/L; and Q4: 0.56–13.03 ug/L); Lead quartile (Q1: 0.05–0.63 ug/L; Q2: 0.64–1.00 ug/L; Q3: 1.01–1.59 ug/L; and Q4: 1.60–61.29 ug/L); Mercury quartile (Q1: 0.11–0.41 ug/L; Q2: 0.42–0.79 ug/L; Q3: 0.80–1.62 ug/L; and Q4: 1.63–50.81 ug/L); Manganese quartile (Q1:1.57–7.43 ug/L; Q2: 7.44–9.24 ug/L; Q3: 9.25–11.55 ug/L; and Q4: 11.56–57.77 ug/L); Selenium quartile (Q1: 85.15–177.87 ug/L; Q2: 177.88–192.47 ug/L; Q3: 192.48–207.54 ug/L; and Q4: 207.55–734.80 ug/L)

*Abbreviations*: *Serum 25(OH)D* 25-hydroxyvitamin D, *CVD* cardiovascular disease

^*^P < 0.05

^**^P < 0.01

^***^P < 0.001

### Serum 25(OH)D mediates the association of heavy metals levels with CVD

**Fig. 5:**
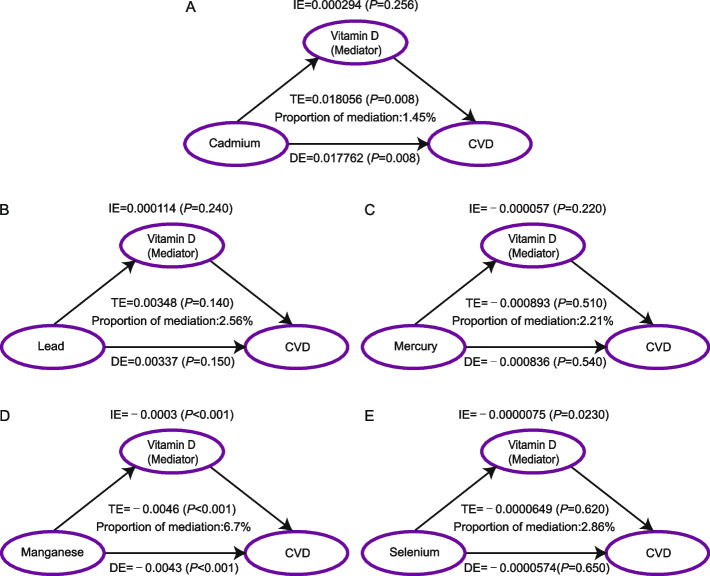
Mediation analysis of serum 25(OH)D on the interaction between heavy metals and CVD. **A** Mediation models of 25(OH)D, Cadmium, and CVD: direct effect (TE = 0.018056; *P* = 0.008) of 25(OH)D (exposure) toward CVD (outcome), and 25(OH)D medication proportion is 1.45%; indirect effect (IE = 0.000294; *P* < 0.001) of 25(OH)D (exposure) toward 25(OH)D (mediator) and effect CVD (DE = 0.017762; *P* < 0.001), from 25(OH)D (mediator) toward CVD (outcome). **B** Mediation models of 25(OH)D, Lead, and CVD: direct effect (TE = 0.00348; *P* = 0.140) of 25(OH)D (exposure) toward CVD (outcome), and 25(OH)D medication proportion is 2.56%; indirect effect (IE = 0.000114; *P* < 0.240) of 25(OH)D (exposure) toward 25(OH)D (mediator) and effect CVD (DE = 0.00337; *P* < 0.150), from 25(OH)D (mediator) toward CVD (outcome). **C** Mediation models of 25(OH)D, Mercury, and CVD: direct effect (TE = -0.000893; *P* = 0.510) of 25(OH)D (exposure) toward CVD (outcome), and 25(OH)D medication proportion is 2.21%; indirect effect (IE = −0.000057; *P* < 0.220) of 25(OH)D (exposure) toward 25(OH)D (mediator) and effect CVD (DE = −0.000836; *P* < 540), from 25(OH)D (mediator) toward CVD (outcome). **D** Mediation models of 25(OH)D, Manganese, and CVD: direct effect (TE = −0.0046; *P* < 0.001) of 25(OH)D (exposure) toward CVD (outcome), and 25(OH)D medication proportion is 6.7%; indirect effect (IE = −0.0003; *P* < 0.001) of 25(OH)D (exposure) toward 25(OH)D (mediator) and effect CVD (DE = −0.0043; *P* < 0.001), from 25(OH)D (mediator) toward CVD (outcome). **E** Mediation models of 25(OH)D, Manganese, and CVD: direct effect (TE = −0.0000649; *P* < 0.620) of 25(OH)D (exposure) toward CVD (outcome), and 25(OH)D medication proportion is 6.7%; indirect effect (IE = −0.0000075; *P* = 0.023) of 25(OH)D (exposure) toward 25(OH)D (mediator) and effect CVD (DE = −0.0000574; *P* = 0.650), from 25(OH)D (mediator) toward CVD (outcome). Abbreviations: 25(OH)D, 25-hydroxyvitamin D; CVD, Cardiovascular disease

We conducted mediation analysis to evaluate whether the association between heavy metals exposure and CVD was mediated by 25(OH)D. The mediating effect of 25(OH)D was not demonstrated in the association between the level of Cadmium, Lead, Mercury, as well as Selenium exposure and CVD (Fig. 5A, B, C, and E). The 25(OH)D level was estimated to explain 6.7% of the association between the Manganese exposure and CVD (Indirect effect (IE): β =  − 0.0003; *P* < 0.001; Total effect (TE): β =  − 0.0046; *P* < u0.001; Direct effect (DE): β =  − 0.0043; *P* < 0.001) (Fig. 5D).

## Discussion

In this study, we examined the mediation of cardiovascular toxicity by 25(OH)D in the general population of the U.S. for the first time. First of all, we found that there was a positive linear correlation between Cadmium, Lead and the prevalence of CVD, a linear negative correlation between Mercury, Manganese and the prevalence of CVD, and a similar L-shaped and U-shaped relationship between Selenium, 25(OH)D and the prevalence of CVD. In addition, we also found that serum 25(OH)D was independently related to Cadmium and Lead. Furthermore, we found a 6.7% association between Manganese levels and CVD prevalence mediated by 25(OH)D levels.

Due to the rapid industrialization of the world, the harmful effects of environmental pollution, which is closely related to industry, have been widely concerned. As an important part of industry-related environmental pollution, heavy metals, including lead, manganese and cadmium, are widespread in the surrounding environment. As a result, the cytotoxicity and tissue toxicity caused by heavy metals has become a major health problem in environmental science [9]. For example, Domingo-Relloso et al. found that urine Cu, Zn, Sb, Cd, Cr and V were independently related to the risk of cardiovascular events in a cohort study of 1,171 Spaniards without CVD, while the urine metals in the mixture were also commonly related to CVD [20]. Another systematic review and meta-analysis involving 348,259 participants also showed that lead, arsenic, copper and cadmium were independently related to the increased risk of CVD, coronary heart disease and stroke, but mercury was not significantly related to these diseases [21]. In addition, Choi et al. also proved in Korea National Health and Nutrition Survey that the blood Cd, Hg and Pb levels of the general population in Korea were strongly correlated with the 10-year CVD risk score, and this correlation was stronger among men [22]. In addition to CVD, Guo et al. found that lead, cadmium and arsenic were closely related to all-cause, CVD-related and cancer-related mortality in a systematic review and meta-analysis of 42 cohort studies, but found no significant correlation between other heavy metals and CVD [23]. However, the above studies have not evaluated the effects of manganese exposure on CVD. In the previous published research, blood manganese levels were negatively correlated with CVD risk based on the published NHANES data [5]. Nevertheless, the mechanism of the association between heavy metals and the risk of CVD is very complicated. Increasing our understanding of the relationship between occupational exposure to heavy metals and CVD risk will require further evaluation of the effects of heavy metal exposure on CVD risks and possible mechanisms. In view of this, Zhu et al. conducted a cross-sectional study of 3081 adults and explored the possible mediating effects of heavy metals on CVD and found that elevated urinary iron, zinc and antimony concentrations were associated with an increased 10-year risk of CVD and that their associations were mediated by mean platelet volume by 17.55%, 6.15% and 7.38%, respectively [24]. Similarly, we found that the association between heavy metals and CVD could be mediated by other factors, that is, 25(OH)D mediated 6.7% of the association between Manganese exposure and CVD risk, but no such interaction between other heavy metals and 25(OH)D was found. These data reveal the relationship between heavy metal exposure and 25(OH)D levels. The relationship between cardiovascular toxicity of heavy metals and CVD is very complicated. Observational studies are insufficient to report the association between heavy metal exposure and CVD. Similar to our study, Yin et al. also found the mediating effect of 25(OH)D by using the same open database, that is, they found that 25(OH)D mediated the negative correlation between acrylamide and obesity in a study containing 10,377 participants from NHANES [25]. As we all know, vitamin D is a fat-soluble vitamin, which can not only exist naturally in food, but also can be used as a dietary supplement to provide nutritional support to people with vitamin D deficiency. Previous studies have shown that lower vitamin D levels are associated with higher prevalence of obesity, cancer, CVD and mortality [13, 26–28]. Our study found that 25(OH)D levels were not only associated with CVD, but also closely related to blood manganese levels. Therefore, in the mediating effect analysis, we further found that 25(OH)D as an intermediary variable was involved in the association between manganese exposure and CVD. This is the first time that we have assessed the regulatory effect of 25(OH)D on heavy metal-induced toxicity in the general population, and the findings of our study will contribute to the pathophysiological understanding of heavy metal toxicity in humans. In addition, our study also provided information for potential interventions in clinical observation studies.

Although we have found for the first time that 25(OH)D mediated a link between Manganese exposure and CVD, the mechanism is still unknown. At present, there may be several potential mechanisms that play a direct or indirect role. For example, there is evidence that after exposure to heavy metals, oxidative stress promotes the transduction of harmful signals and leads to CVD [9], while Christakos et al. have shown that a certain level of vitamin D can resist oxidative stress and reduce the risk of CVD [29]. Therefore, we hypothesized that 25(OH)D can reduce the oxidative stress induced by heavy metal exposure and reduce the prevalence of CVD. In addition, it is reported that heavy metal exposure can enhance the inflammatory response, which plays a vital role in the pathogenesis of CVD [30, 31]. However, Guillot et al. reviewed vitamin D and inflammation and found that the immunomodulatory effect of vitamin D is not only suitable for rheumatoid arthritis and inflammatory bowel disease, but also for infection and CVD [32]. Therefore, we believed that 25(OH)D can regulate the inflammatory activity caused by heavy metals and thus reduce the risk of CVD. Previously, most studies mainly focused on the relationship between vitamin D, heavy metal exposure and CVD, rather than their interaction, while the level of serum 25(OH)D can mediate the association between heavy metal exposure and CVD in our study. Mediating factors can help to sort out the biological pathways that link harmful factors with cardiovascular events, which is essential for which is essential for detecting and preventing cardiovascular events at an early stage.

Although our study has achieved encouraging results, there were still several limitations. First, as a cross-sectional study, we only conducted a single assessment of heavy metal exposure and did not continuously monitor long-term exposure to heavy metals and their longitudinal effects on CVD. Second, instead of real medical records, the self-reported approach was used to assess the incidence of CVD among participants. This may have led to a bias in the results. Third, this study was a cross-sectional study. Therefore, the causal relationship between heavy metal exposure and CVD cannot be determined. Additionally, although the intermediary effect analysis well described the effects of heavy metals on CVD, it was still unable to determine the causal correlation. Forth, there might be other potential influencing factors that have not been adjusted, such as environment, diet and genetic susceptibility. Fifth, most of the heavy metals are excreted from the kidneys, so the levels of heavy metals in the urine may better reflect their long-term exposure, while we only assessed the blood levels of heavy metals. Finally, our study only covered the general population in the United States, so the results may not be extended to other countries and populations. Therefore, further studies are necessary to determine whether heavy metal exposure and 25(OH)D interact.

## Conclusions

According to our study, there was a substantial inverse correlation between Manganese exposure and 25(OH)D. In addition, 25(OH)D mediated a negative connection between manganese and incident CVD shows that 25(OH)D supplementation may enhance the protective effect of manganese intake on CVD. An in-depth study of Manganese’s relationship to serum 25(OH)D is required.

## Data Availability

Survey data is available for data consumers and researchers all across the globe on the internet (https://www.cdc.gov/nchs/nhanes/).
